# From Safe to Stranded: Land Use and Climate Change Threaten Habitat of Iconic Australian Macropods

**DOI:** 10.1002/ece3.72236

**Published:** 2025-10-09

**Authors:** Elizabeth Ann Brunton, Aaron James Brunton, Gabriel Conroy

**Affiliations:** ^1^ School of Science, Technology and Engineering University of the Sunshine Coast Sippy Downs Queensland Australia; ^2^ Centre for Bioinnovation University of the Sunshine Coast Sippy Downs Queensland Australia

**Keywords:** climate refugia, habitat fragmentation, kangaroo, long nosed potoroo, pademelon, urban ecology, wallaby

## Abstract

As the footprint from human population increases, the associated modification and conversion of natural landscapes in a changing climate places significant pressure on terrestrial wildlife. Since areas of high biodiversity are most affected by urbanisation, there is a need to identify future challenges for species in these regions in the context of intensifying climate change. We investigated habitat dynamics for seven Macropodid species found in the rapidly urbanising, biodiverse Southeast Queensland (SEQ) region of Australia. Habitat suitability was modelled using presence‐only occurrence data (2000–2023) in combination with bioclimatic and landscape variables. We employed a balanced Random Forest algorithm to model species distributions, project current and potential habitat and identify key bioclimatic and landscape factors influencing conservation management. A greater amount of predicted current suitable habitat (over one third) for eastern grey kangaroos, swamp wallabies and red‐necked wallabies is within the urban footprint, than in protected areas. Conversely, most current suitable habitats for the other species were predicted to occur in protected areas. Worryingly, a decline in suitable habitat (83%–96% reduction) is projected for all seven species under future climate scenarios. Our results reveal the vulnerability of macropods in the region which face compounded threats from urbanisation and climate‐induced habitat loss. This study's findings highlight a complex set of factors that could hinder macropod species' adaptability to future environmental changes, elevating ‘least concern’ species to ‘of concern’. Combined pressures from climate change, urbanisation and habitat loss necessitate a broad, adaptive approach to wildlife conservation in human‐dominated landscapes.

## Graphical abstract

Habitat suitability mapping for macropods under current and future climate scenarios reveals critical areas of conservation concern.

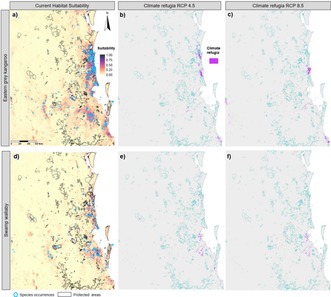

## 1 Introduction

As the footprint from a growing human population continues to grow in magnitude and intensity, the associated modification of natural systems to accommodate built infrastructure for increasing human demand is placing significant pressure on wildlife worldwide (Simkin et al. 2022). Urbanisation impacts are often more pronounced in areas of higher biodiversity since many global biodiversity hotspots occur in areas with high human population densities, therefore compounding the magnitude of potential urbanisation impacts (Myers et al. 2000). While the potential implications of urbanisation and climate change on biodiversity have been extensively studied, they are often examined in isolation. For example, urban planning that incorporates biodiversity considerations typically focuses on retaining remnant ecosystems and protecting habitats suited to current climate conditions. Yet, the combined effects of climate and land use change may have a complex and detrimental impact on terrestrial mammal species globally (Haight et al. 2023). In a recent study modelling future species distributions of more than 2000 animal species in North American cities, Filazzola et al. (2024) predicted high rates of species losses in urban areas, indicating that mitigation strategies in urban areas are needed that consider potential future changes to urban wildlife habitat that incorporate climatic and land use change. Since urban biodiversity is of growing importance to global conservation efforts (Knapp et al. 2021), the need to understand and consider the extent and potential interactions of climate change and urbanisation for effective urban biodiversity conservation is highlighted (Urban et al. 2024). While the link between urbanisation and climate change has been well explored in relation to social and economic impacts of climate change (Garschagen and Romero‐Lankao 2015), it is not well considered in wildlife conservation literature. There is, however, a growing acknowledgement of the value of identifying climate refugia to inform conservation management, as a potential means to retain biodiversity and ecosystem function in rapidly changing environments (Morelli et al. 2020). Climate refugia can be generally considered as areas that species may retreat to and persist in under changing climatic and environmental conditions (Keppel et al. 2012). The mapping of climate refugia can therefore provide a key link between spatial planning information and species distributions and their vulnerability to future change, to aid in conservation prioritisation and species management (Buenafe et al. 2025; Morelli et al. 2025).

In this context, understanding the impacts of climate change on Australia's unique fauna is crucial. Macropodiformes is a diverse suborder which contains iconic species (e.g., kangaroos and wallabies) that occur across the Australian continent and in many different ecosystem types, with many macropod species distributions strongly shaped by rainfall and temperatures (Johnson et al. 1989; Ritchie and Bolitho 2008; Southwell et al. 1999). This suborder includes the Macropodidae, Potoridae and Hypsiprymnodontidae families (Phillips et al. 2023), which collectively contain 23 species listed as threatened federally. Threatened macropod species are mostly in smaller weight classes; the larger macropod species in Australia have widespread ranges and are not classified as of conservation concern nationally (Australian Government 2025). Yet, there is growing evidence that these larger macropods can be negatively impacted by land use modification (Reid et al. 2020), especially due to urbanisation, with meta‐populations from some regions being decimated from habitat loss and increases in road density and traffic volumes (Brunton, Srivastava, and Burnett 2018; Bond and Jones 2014) and associated with human population growth (Brunton, Srivastava, Schoeman, and Burnett 2018; Brunton et al. 2022; Herbert et al. 2021). This is particularly true for those species and/or populations that inhabit urban and peri‐urban areas (Herbert et al. 2021; Coulson et al. 2014).

Recent studies have used climatic data to predict climate refugia to guide conservation priorities for species in fragmented landscapes (Mulhall et al. 2023; de Silva et al. 2023). Previous research by Ritchie and Bolitho (2008) modelled climatic conditions to assess future distributions of four large macropodids in northern Australia under different future climate scenarios and highlighted the importance of climatic gradients and seasonality for macropod distribution. All four species were projected to undergo range restrictions under future climate scenarios, with the common wallaroo having the broadest climatic envelope. In Queensland, the common wallaroo was more likely to occur in tropical climates than the eastern grey kangaroo, which was more associated with subtropical climates, with their climatic range restricted to the east of Queensland. Notably, this study did not incorporate land use change, and therefore, the suitable habitat available to macropods in urban areas is likely to be less than projected. In Queensland, macropod populations with home ranges occurring in areas with high human populations will not only face potential range restrictions due to climate change but also increasing fragmentation and loss of what suitable habitat remains.

The Southeast Queensland region of Australia is an area of high biodiversity that has experienced rapid land use change and urbanisation in previous decades (Simmons et al. 2018). More than 800 vertebrate species occur in the region; however, biodiversity is threatened due to rapid human population growth, unsustainable land management practices and native vegetation clearing and fragmentation (EHP 2016). Macropodid species within the region face significant pressures from habitat loss and road mortality (Brunton, Srivastava, and Burnett 2018) because of a rapidly expanding human population and infrastructure footprint (Brunton et al. 2022; Taylor‐Brown et al. 2019).

Recent increases in macropod road mortalities and population declines and planned human population and infrastructure growth raise questions about the sustainability of the region's macropod populations under uncertain land use and climatic change. Therefore, in this study, we investigated the habitat dynamics for seven macropodid species found in the Southeast Queensland region to assess (i) current extent of habitat suitability, (ii) protection status of high suitability habitat and (iii) how habitat suitability may change under future climate scenarios. To achieve this, we applied species distribution models, which are an effective and commonly applied tool in conservation to inform conservation planning (Southwell et al. 2024; Elith and Leathwick 2009), and to predict responses of species to future change, for example, climate (Haight et al. 2023; Franklin 2013) and land use change (Mulhall et al. 2023; de Silva et al. 2023).

## 2 Methods

### 2.1 Study Region and Species

**FIGURE 1:**
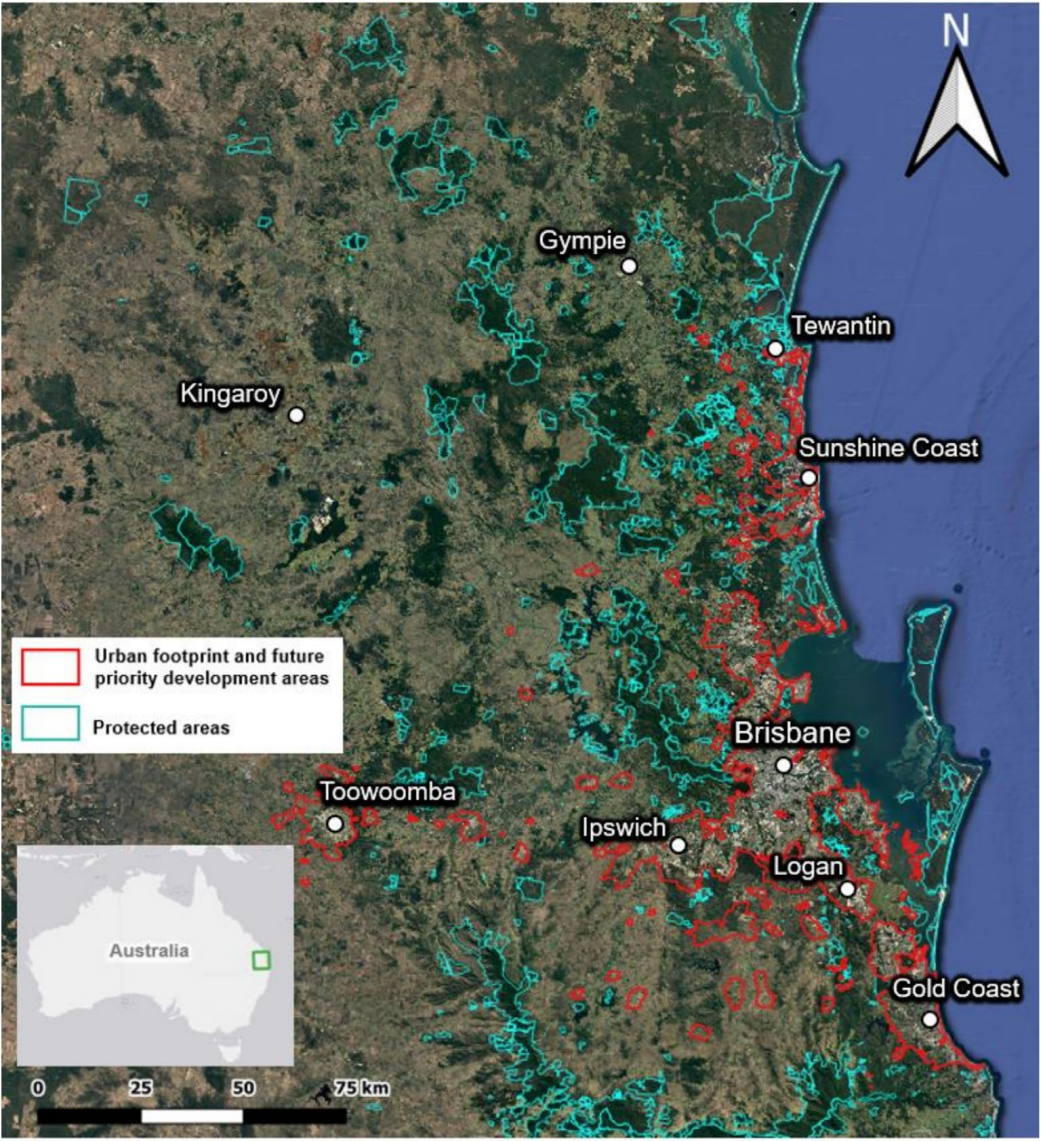
Map of study region in Southeast Queensland, Australia, highlighting extent of protected areas and urban footprint which encompasses current urban infrastructure as well as planned urban expansion to 2048.

The Southeast Queensland region of Australia is highly diverse, with 92% of the SEQ bioregion noted as having state or regionally significant biodiversity values. The climate is largely subtropical and contains highly urbanised areas as well as significant natural areas, including the World Heritage Listed Gondwana Rainforests of Australia (Williams et al. 2011). Land use types include agriculture, forestry, urban infrastructure, modified green spaces and a matrix of remnant ecosystems including coastal heath and wetlands, rainforests and wet and dry sclerophyll forests (Figure 1). This recognised biodiversity hotspot includes some of the last remaining fragments of subtropical rainforest that once extensively covered the region (Braithwaite et al. 2010). Additionally, the Gondwana Rainforests serve as critical habitat for numerous threatened plant and animal species and are under significant threat from climate change (Osipova et al. 2017).

This study considers seven macropodid species found in the Southeast Queensland region. Six of the seven species are currently classified as ‘least concern’ in Queensland: Eastern grey kangaroo ( *Macropus giganteus* ), swamp wallaby ( *Wallabia bicolor* ), red‐necked wallaby (*Notamacropus rufogriseus*), red‐necked pademelon ( *Thylogale thetis* ), red‐legged pademelon ( *Thylogale stigmatica* ) and black‐striped wallaby (*Notamacropus dorsalis*). The other study species, long‐nosed potoroos ( *Potorous tridactylus* ), are classified as vulnerable in Queensland (Queensland Government 2023) and New South Wales (NSW Government 2024).

### 2.2 Occurrence Data Collection & Screening

In this study, a total of 5025 georeferenced records were used from a variety of resources across south‐eastern Queensland, Australia (Figure 1). Live occurrence data were collected for seven macropod species (Table 1). Occurrence records included verified or validated data from the most recent 25 years (January 2000 to June 2024). Unpublished data were accessed from a variety of sources that had been obtained for previous research and management objectives. Occurrence records were also downloaded from a publicly accessible iNaturalist project (iNaturalist 2024), Atlas of Living Australia website (ALA 2024), applying the following exclusion criteria: spatially suspect records (coordinates missing or invalid), duplicate records and all records where the outlier layer count is three or more; records with no image attached or no validation or verified identification. The final dataset (Table 1) only included animal occurrences with ≤ 100 m accuracy documented through sightings, field observations, camera traps or other non‐roadkill methods.

**TABLE 1:** Macropod species, conservation status and sample sizes used for species distribution modelling for the Southeast Queensland region.

Species	Scientific name	Conservation status in study area (QLD)	Conservation status in state adjacent to study area (NSW)	Occurrence records (*n*)
Eastern grey kangaroo	*Macropus giganteus*	Least concern	Least concern	2465
Swamp wallaby	*Wallabia bicolor*	Least concern	Least concern	600
Red‐necked wallaby	*Notamacropus rufogriseus*	Least concern	Least concern	911
Red‐necked pademelon	*Thylogale thetis*	Least concern	Least concern	208
Red‐legged pademelon	*Thylogale stigmatica*	Least concern	Vulnerable	166
Black‐striped wallaby	*Notamacropus dorsalis*	Least concern	Endangered	134
Long‐nosed potoroo	*Potorous tridactylus*	Vulnerable	Vulnerable	62

Abbreviations: NSW = New South Wales; QLD = Queensland.

### 2.3 Model Predictors

All climate and environmental gridded variables were obtained via the CSIRO Data Access Portal (http://datanet.csiro.au/dap—accessed on 4 June 2024) and EcoCommons data repository (https://www.ecocommons.org.au/data‐analysis/—accessed on 4 June 2024). For current projections, we used the 1990–2005 climate dataset comprised of nine variables: TNM (mean annual minimum temperature), TXM (mean annual maximum temperature), TXX (annual maximum of daily maximum temperature), TXI (mean minimum monthly maximum temperature), TNI (mean minimum monthly minimum temperature), TNX (mean maximum monthly minimum temperature), PTA (average total annual rainfall), PTX (mean maximum monthly rainfall) and PTI (mean minimum monthly rainfall). In addition, we tested five environmental variables based on factors known to be of ecological relevance to macropod species (Johnson et al. 1989; Ritchie and Bolitho 2008; Southwell et al. 1999; Urbanek et al. 2025): Australia NDVI—normalised vegetation index 2018–2019 (NDVI), elevation—Digital Elevation Model version 3 2008, soil—soil and landscape grid national soil attribute maps, tree canopy cover—Australia Forest Change, Tree Canopy Cover 2000 (tree cover) and moisture—annual mean moisture index (Bio28). All variables were resampled using bilinear interpolation to align data to 9‐arcsecond resolution (250‐m^2^ grids).

To minimise the potential of model overfitting, multicollinearity among predictor variables was assessed using variance inflation factors (VIF). The VIF values were calculated through the vifcor function from the R package *usdm* (Naimi 2015), identifying pairs of highly correlated variables, with those exceeding a threshold of 0.7 removed from the analysis. This stepwise approach allowed for the exclusion of variables with the highest linear correlations. Of the initial 14 variables, seven were identified as collinear and excluded, leaving seven environmental predictors: moisture, NDVI, soil, tree cover, mean maximum monthly maximum temperature and mean maximum monthly rainfall.

### 2.4 Distribution Modelling and Interpretation

All models were constructed and visualised in the R environment (R Team 2020), using the packages ‘SDM’ (Naimi and Araújo 2016), terra (Hijmans et al. 2022) and tmap (Tennekes 2018). Macropod distributions were initially modelled using four SDM techniques including generalised linear models (GLM), random forest (RF), MaxEnt and boosted regression trees (BRT) to determine which methods yielded the highest predictive accuracy (Barbet‐Massin et al. 2012). RF and MaxEnt models were the top two performing models across all species and therefore applied to all further analyses (Table S1).

We used tuned parameters for each model to optimise model accuracy while reducing the potential for overfitting, and to maintain computational efficiency. For the RF and MaxEnt models, we used a random data partitioning approach with 70% training data and 30% for the validation (test) set method. In addition to RF models, we applied *n* = 500 trees as well as a downsampling, otherwise termed ‘balanced’ background sampling method with the model (Chen et al. 2004). This balanced method can significantly enhance the predictive accuracy of RF‐based SDMs (Evans and Cushman 2009; Freeman et al. 2012). In essence, downsampling mitigates overfitting by fitting shallower decision trees than the default RF configuration and balancing the training dataset, akin to equal‐sampling RFs. This technique subsamples the majority class (background data) for each tree, resulting in faster prediction implementation while maintaining a more comprehensive representation of the environmental space in the study region (Valavi et al. 2021; Freeman et al. 2012). In the MaxEnt models, we applied a combination of linear and quadratic sampling along with regularisation parameters (beta = 2, lambda = 0.5). Finally, all models were evaluated with a five *k*‐fold cross‐validation approach, with all metrics presented averaged across the five cross‐validations for each model.

Model accuracy was evaluated from training datasets to calculate several threshold‐independent measures of predictive performance: (1) the average area under the receiver operating characteristic curve (AUC_ROC_), abbreviated here to AUC; (2) Pearson correlation (COR) which represents model performance and power to discriminate between suitable and unsuitable habitat; (3) true skill statistic (TSS), a metric to assess model accuracy considering both sensitivity (true positive rate) and specificity (true negative rate); (4) and (5) deviance (Dev), which measures the goodness of fit of the model (Table S1).

To generate distribution models of the Macropod species under current conditions, model‐averaged parameter estimates were then used to generate class‐probability maps on a continuous scale from 0 to 1 at 250‐m^2^ resolution.

### 2.5 Background Data Selection

For RF models, we used a presence‐only approach to construct SDMs, with background data covering pseudoabsence points across the study domain as absences. Although presence‐only data can pose challenges for some modelling approaches, RF models have demonstrated strong predictive performance when class imbalance and environmental overlap are appropriately managed, making them a viable option for species distribution modelling in the absence of reliable absence data (Valavi et al. 2021).

Pseudoabsences were constructed for each taxa using the ‘eDist’ method. This approach uses a random sampling weighted by environmental distance, which involves randomly selecting locations across a geographic area (study domain) but assigns greater weight to locations with environmental conditions that are more dissimilar to those where the species have been observed (Naimi and Araújo 2016). By applying this method of background data selection, to the SDMs of macropods across a rapidly urbanising yet, highly heterogeneous landscape ensures a diverse representation of environmental conditions in the background data, improving the model's robustness and accuracy (Naimi and Araújo 2016). It is important to acknowledge that the number of background data points remains an ongoing challenge to SDMs. However, for this research, we considered the recent evidence to support that a minimum of 10,000 background samples is required to represent all environments across a study domain (Valavi et al. 2022; Barbet‐Massin et al. 2012). Hence, we concluded that *n* = 10,000 was a sufficient number of pseudoabsences (RF models) and background points (MaxEnt) to achieve representation of variation in the environment for the study domain.

### 2.6 Future Projections

Projections of macropod habitat suitability under future climate scenarios were performed using a set of 9‐s gridded climate change variables from two general circulation models: GFDL ESM2 and ACCESS 1.0, obtained from the CSIRO Data Access Portal (http://datanet.csiro.au/dap—accessed on 4 June 2024). These models were selected to balance a global perspective (Dunne et al. 2013) with region‐specific accuracy for Australia (Bi et al. 2013). To enhance the robustness of the future projections, we used an ensemble approach by calculating the mean of the two climate models using the overlay (…, fun = mean) function in R. This simple ensemble mean allowed us to account for uncertainty and variability between the models and produce a single, representative projection of future suitability.

This future model was used to generate projections based on two emissions scenarios, the representative concentration pathways (RCP) 4.5 (moderate mitigation) and RCP 8.5 (high emissions), for the year 2070. To visualise future projections under the two emission scenarios, we applied a threshold approach to convert continuous habitat suitability values (ranging from 0 to 1) into binary maps, where suitable habitat is classified as ‘present’ (1) and unsuitable habitat as ‘absent’ (0). While various methods exist for converting continuous probability maps into binary classifications, using a fixed threshold (e.g., > 0.5) is among the least reliable approaches (Liu et al. 2005). Instead, we applied a percentile threshold method, specifically the 10th percentile of training presence (P10), to classify habitat suitability in the binary maps. This approach excludes habitat suitability values lower than the probability threshold of the lowest 10% of occurrence records (Guillera‐Arroita et al. 2015). Areas with suitability above this threshold (i.e., ≥ P10) were considered more likely than not to support macropods under future climates and are therefore referred to as ‘climate refugia’. We also generated the full set of continuous probability maps (Figures S1–S4) to visualise the spatial patterns of habitat suitability in greater detail. We also generated variable importance plots (Table S2) to summarise the relative contribution of each predictor to model performance.

### 2.7 Static Environmental Predictors

Environmental predictors elevation, soil type, NDVI and treecover were treated as static across future climate scenarios. This decision reflects both practical and ecological considerations. Many of these variables change only over geological timescales (e.g., soil type) or lack reliable future projections (e.g., elevation and vegetation cover), making it necessary to hold them constant in climate‐based species distribution models (Austin 2002; Stanton et al. 2012). Previous studies have shown that including static variables alongside dynamic climate predictors can improve model performance, especially when interactions exist between them (Stanton et al. 2012). While this introduces the assumption that these variables remain unchanged, it is considered preferable to excluding them altogether, as they often represent key ecological constraints on species distributions. This approach is consistent with previous work in the region, including our own modelling of plant distributions under climate change (Brunton et al. 2023), where static predictors were retained to preserve ecological realism and model integrity.

### 2.8 Future Land Use and Climate Impacts on Habitat Protection

Urban footprints and protected area layers were overlaid with current high suitability habitat for each species and future projected high suitability habitat to estimate the area of high suitability habitat within the urban footprint and protected areas. We quantified the footprint of urban infrastructure and growth using the ‘Urban Footprint’ regional land use category as mapped in the Southeast Queensland Regional Plan 2023 (Queensland Government 2023). This land use mapping encompasses current urban infrastructure as well as planned urban expansion to 2048, and 14% of the region is categorised as ‘Urban Footprint’. Protected areas were quantified by utilising the terrestrial layer of protected areas for the study region, sourced from the Collaborative Australian Protected Area Database (CAPAD 2020). CAPAD (2020) provides comprehensive data on all types of protected areas in Australia, including federal, state and private reserves, as well as land parcels designated for future protection. The shapefile was imported into R and filtered to only include protected areas where the focal species occur.

## 3 Results

### 3.1 Current Suitable Habitat

**FIGURE 2:**
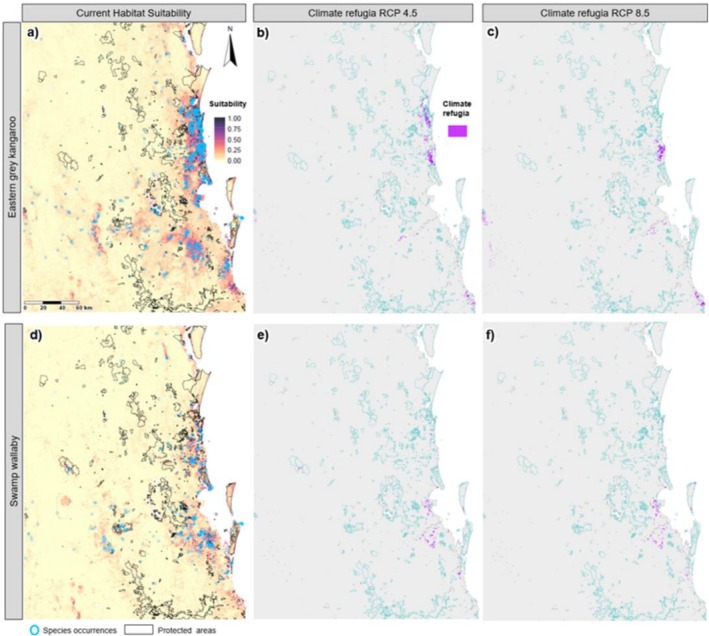
Predicted species distributions for the eastern grey kangaroo ( *Macropus giganteus* ) and swamp wallaby ( *Wallabia bicolor* ), modelled using random forest. Panels (a–c) shows habitat suitability for the eastern grey kangaroo: (a) current distribution, (b) climate refugia projected for 2070 under the moderate representative concentration pathway (RCP) (RCP 4.5) and (c) climate refugia under the high RCP for 2070 (RCP 8.5). Panels (d–f) depict the same conditions for the swamp wallaby: (d) current distribution, (e) climate refugia for 2070 under the moderate RCP (RCP 4.5) and (f) climate refugia under the high RCP (RCP 8.5). Climate refugia were defined as areas (highlighted in pink) of habitat that will remain suitable under future climate conditions based on the probability of occurrence ≥ P10 threshold for each species. Aqua outlines represent protected areas.

The seven macropod species were distributed patchily across the study area, with three species having the largest area of suitable habitat: eastern grey kangaroo, swamp wallaby and red‐necked wallaby, respectively (Table 2). Suitable habitat for red‐necked wallabies has both a coastal and inland distribution, whereas eastern grey kangaroo and swamp wallaby suitable habitat is predominantly on the coastal plains (Figures 2 and 4). There is low overlap (< 3%) between eastern grey kangaroo suitable habitat area and that of other macropod species' habitat extent, except for swamp wallabies (15% overlap) (Table 3). Approximately 35% of swamp wallaby habitat overlaps with eastern grey kangaroo habitat, with 20% overlapping with red‐necked wallaby habitat, and negligible overlap with other species. Red‐necked wallabies have little overlap with the remaining macropod species (Table 3).

**TABLE 2:** Current and future predicted extents of suitable habitat for seven macropod species in the Southeast of Queensland, Australia.

Species name	Current suitable habitat (total area, km^2^)	Future suitable habitat R45 (total area, km^2^)	Future suitable habitat R85 (total area, km^2^)
*Eastern grey kangaroo*	1403.60	190.50 (−86.43)	148.40 (−89.43)
*Swamp wallaby*	581.90	92.60 (−84.09)	76.10 (−86.92)
*Red‐necked wallaby*	512.10	35.60 (−93.88)	79.50 (−84.48)
*Red‐legged pademelon*	152.20	24.80 (−83.71)	24.40 (−83.97)
*Red‐necked pademelon*	119.10	17.00 (−85.72)	18.50 (−84.47)
*Black‐striped wallaby*	16.80	1.90 (−88.69)	0.70 (−95.83)
*Long‐nosed potoroo*	58.10	4.70 (−91.91)	5.10 (−91.22)

*Note:* Future climate scenarios: R45 (moderate) and R85 (extreme). Percentage change from current suitable habitat shown in brackets.

**TABLE 3:** Habitat area (km^2^) overlap matrix for seven macropod species in Southeast Queensland.

Species	M. gig	W. bic	N. Ruf	P. tri	T. sti	T. the	N. dor
M. gig		206.80	38.90	0.10	0.20	0.00	0.10
W. bic	206.80		118.00	0.20	0.20	0.20	1.90
N. Ruf	38.90	118.00		5.00	2.60	8.10	8.80
P. tri	0.10	0.20	5.00		12.20	36.40	0.00
T. sti	0.20	0.20	2.60	12.20		46.20	0.00
T. the	0.00	0.20	8.10	36.40	46.20		0.10
N. dor	0.10	1.90	8.80	0.00	0.00	0.10	

Abbreviations: M. gig = Eastern grey kangaroo; N. dor = Black‐striped wallaby; N. ruf = Red‐necked wallaby; P. tri = Long‐nosed potoroo; T. sti = Red‐legged pademelon; T. the = Red‐necked pademelon; W. bic = Swamp wallaby.

**FIGURE 3:**
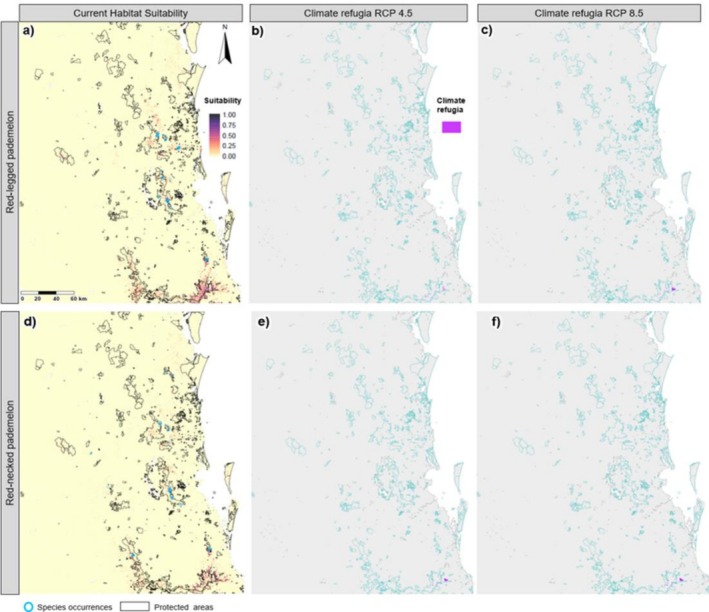
Predicted species distributions for the red‐legged pademelon ( *Thylogale stigmatica* ) and red‐necked pademelon ( *Thylogale thetis* ), modelled using random forest. Panels (a–c) show habitat suitability for the red‐legged pademelon: (a) current distribution, (b) climate refugia projected for 2070 under the moderate representative concentration pathway (RCP) (RCP 4.5) and (c) climate refugia under the high RCP for 2070 (RCP 8.5). Panels (d–f) depict the same conditions for the red‐necked pademelon: (d) current distribution, (e) climate refugia for 2070 under the moderate RCP (RCP 4.5) and (f) climate refugia under the high RCP (RCP 8.5). Climate refugia were defined as areas (highlighted in pink) of habitat that will remain suitable under future climate conditions based on the probability of occurrence ≥ P10 threshold for each species. Aqua outlines represent protected areas.

Suitable habitat for both pademelon species has a predominantly inland distribution, with much of the habitat within or adjacent to protected areas. Aside from the most southern part of their distribution, areas of suitable habitat are relatively small and fragmented across the study area (Figure 3, Table 2). Red‐necked pademelons have a large overlap of suitable habitat area with red‐legged pademelons (39%) and long‐nosed potoroos (30%). However, red‐legged pademelon's suitable habitat only overlaps with 8% of the long‐nosed potoroos (Table 3).

**FIGURE 4:**
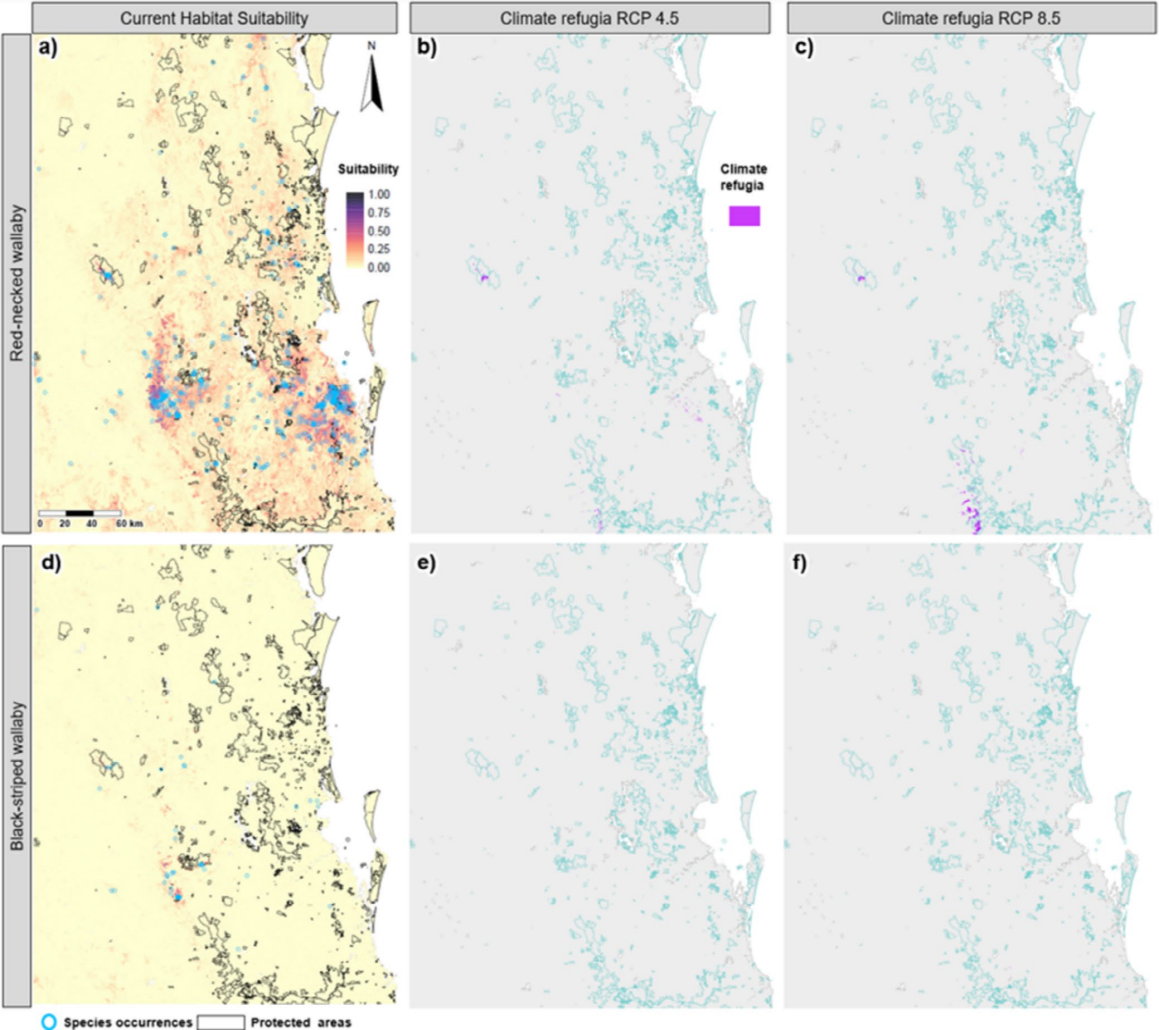
Predicted species distributions for the red‐necked wallaby ( *Macropus rufogriseus* ) and black‐striped wallaby (*Notamacropus dorsalis*), modelled using random forest. Panels (a–c) show habitat suitability for the red‐necked wallaby: (a) current distribution, (b) climate refugia projected for 2070 under the moderate representative concentration pathway (RCP) (RCP 4.5) and (c) climate refugia under the high RCP for 2070 (RCP 8.5). Panels (d–f) depict the same conditions for the black‐striped wallaby: (d) current distribution, (e) climate refugia for 2070 under the moderate RCP (RCP 4.5) and (f) climate refugia under the high RCP (RCP 8.5). Climate refugia were defined as areas (highlighted in pink) of habitat that will remain suitable under future climate conditions based on the probability of occurrence ≥ P10 threshold for each species. Aqua outlines represent protected areas.

Black‐striped wallaby habitat is sparsely and patchily distributed, with the smallest extent of all species. Suitable habitat is predominantly in the bottom half of the study region, with low numbers of occurrence records across the study area (Figure 4, Table 2). Over half of the small area of suitable habitat for the species overlaps with red‐necked wallaby habitat (Table 3).

**FIGURE 5:**
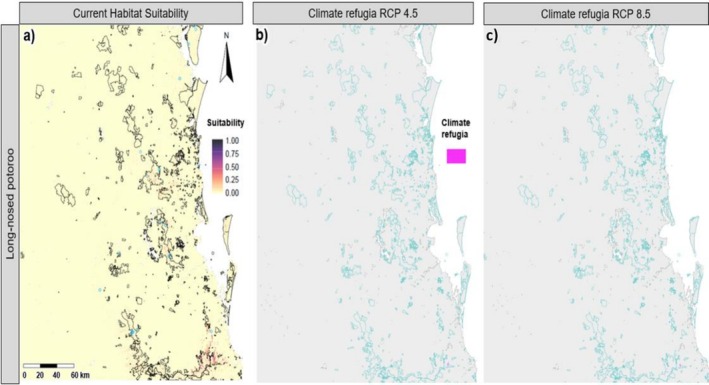
Predicted species distributions for the long‐nosed potoroo (*Potorous tridactylus—*modelled using random forest): (a) current distribution, (b) climate refugia projected for 2070 under the moderate representative concentration pathway (RCP) (RCP 4.5) and (c) climate refugia under the high RCP for 2070 (RCP 8.5). Climate refugia were defined as areas (highlighted in pink) of habitat that will remain suitable under future climate conditions based on the probability of occurrence ≥ P10 threshold for each species. Aqua outlines represent protected areas.

Long‐nosed potoroos also have a sparse and patchy distribution, with most current suitable habitat in the southernmost part of the study area, predominantly in the Main Range and Springbrook National Parks (that are part of the Gondwana Rainforest Network), as well as unprotected areas that straddle the QLD‐NSW border (Table 2, Figure 5). Suitable habitat for both pademelon species overlaps with the long‐nosed potoroos, with 63% of potoroo habitat overlapping with red‐legged pademelons and 21% overlapping with red‐necked pademelons. Habitat overlap with other species is negligible (Table 3).

### 3.2 Future Climate and Land Use Influence and Habitat Protection

#### 3.2.1 Climate Refugia

All seven species are projected to undergo a drastic decrease in suitable habitat (83%–100% reduction) under both future climate scenarios (Table 2, Figures 2, 3, 4, 5). Only small areas of climate refugia are predicted for each species, and for most of the species, the amount of suitable habitat projected to be in protected areas is at least an order of magnitude less under future climate scenarios compared to current conditions (Table 4). For pademelon species, future climate refugia only remain in the southern part of their distributions, mostly in the National Parks and private areas that span the QLD‐NSW border (Figure 3). Black‐striped wallabies and long nosed potoroos have very low predicted habitat areas overall (< 3 km^2^; Figures 4 and 5) (Table 4). For red‐necked and swamp wallabies, most refugia are projected in the southern part of the study area, with eastern grey kangaroos being the only species with future refugia in the northern part of the region (Figures 2 and 4).

**TABLE 4:** Extent of current and future suitable macropod habitat within the urban footprint (UF) and protected area network (PA) in Southeast Queensland.

Species	Current habitat in UF (km^2^)	Current habitat in PA (km^2^)	Future habitat (R45) in PA (km^2^)	Future habitat (R85) in PA (km^2^)
*Eastern grey kangaroo*	641.00 (45.67)	267.00 (19.02)	35.90	15.40
*Swamp wallaby*	246.30 (42.33)	172.10 (29.58)	16.00	11.80
*Red‐necked wallaby*	186.70 (36.46)	67.10 (13.10)	8.00	18.10
*Red‐legged pademelon*	6.90 (4.53)	98.10 (64.45)	18.10	13.90
*Red‐necked pademelon*	9.00 (7.64)	59.30 (49.79)	7.70	8.50
*Black‐striped wallaby*	0.40 (2.38)	5.00 (30.00)	0.90	0.10
*Long‐nosed potoroo*	1.20 (2.10)	30.40 (52.32)	2.40	3.30

*Note:* Percentages of current suitable habitat extent shown in brackets.

#### 3.2.2 Land Use Influence and Habitat Protection

At least one‐third of the current suitable habitat for the larger species; eastern grey kangaroos, swamp wallabies and red‐necked wallabies, is within the urban footprint, which is a greater amount than is predicted for these species in protected areas (Table 4). The opposite scenario is apparent for the remaining species where a greater proportion of habitat is contained within protected areas than in the urban footprint (Table 3). These species also have low proportions (2.1%–7.6%) of their habitat within the urban footprint.

## 4 Discussion

This study has highlighted the significant impacts of land use and climate change on macropodid species in a biodiverse region of Australia, where projections indicate a large reduction in suitable habitat under both moderate and extreme climate scenarios. Our results underscore the vulnerability of key macropod species that are currently considered as ‘least concern’ (in terms of their current conservation status from a legislative perspective), particularly eastern grey kangaroos, swamp wallabies and red‐necked wallabies, which face compounded threats from urbanisation and climate‐induced habitat loss. These species are at an increased risk due to the limited extent of habitat within protected areas within the study region and the overwhelming presence of their habitats within urban environments. Urban landscapes present numerous challenges for wildlife, especially for large herbivores like macropods. The effects of roads, habitat fragmentation and the broader processes of urbanisation on macropod populations have been well documented in previous studies (Brunton, Srivastava, and Burnett 2018; Herbert et al. 2021). As macropods are pushed into lower suitability habitats, particularly in fragmented urban areas, they face heightened risks including physiological stress (Brunton et al. 2020), disease (Brandimarti et al. 2021), higher mortality rates (Brunton, Srivastava, and Burnett 2018; Ramp and Ben‐ami 2006; Taylor‐Brown et al. 2019), a potential decline in genetic diversity (Brunton et al. 2022) and reduced gene flow between populations (Urbanek et al. 2025). These risk factors are likely to exacerbate the challenges macropods in the region already face, contributing to the deterioration of the maintenance of healthy populations over time and potential localised extinctions (Brunton, Srivastava, and Burnett 2018; Ramp and Ben‐ami 2006), given the high proportion of eastern grey kangaroo, swamp wallaby and red‐necked wallaby suitable habitat that occurs within the urban footprint. With urban sprawl continuing in the study region, it is critical that urban planning incorporates wildlife corridors and connectivity to minimise habitat fragmentation and support species movement.

Concerningly, black‐striped wallabies and long‐nosed potoroos were projected to lose the greatest proportion of their already limited suitable habitat. While the long‐nosed potoroo is currently listed as vulnerable in QLD, black‐striped wallabies are not formally recognised as threatened, yet the limited occurrence records and smaller extents of suitable habitat for both species highlight significant conservation risks under future climate scenarios, as well as from other factors such as habitat fragmentation. Habitat loss and fragmentation in the nearby coastal plains of northern NSW due to rapid human development has already reduced potoroo habitat to very few, small, fragmented areas (Andren et al. 2018). The projected loss of suitable habitat under both climate scenarios highlights the importance of their current remaining habitats. This situation indicates a need for renewed conservation attention, including cross‐jurisdictional efforts across state, local government and private land tenures, as the most critical habitats may lie in areas not currently subject to targeted conservation actions (e.g., national parks). The rehabilitation of these habitats, alongside a focus on private land conservation and shared public spaces, could play an important role in supporting the future persistence of these species. In particular, a focus on improving connectivity of protected areas through private land conservation efforts is needed, as long‐nosed potoroo populations already exhibit fine‐scale structuring with regional populations displaying high differentiation (Frankham et al. 2016).

For the pademelon species (red‐necked and red‐legged), private land conservation efforts may also play a key role in conservation by connecting protected areas, since both species currently have a higher proportion of their habitat protected. These species live in rainforest and wet sclerophyll forests and are known to live in sympatry (Jarman and Phillips 1989; Smith et al. 2024). This is supported by the high overlap of suitable habitat predicted between the species in the Southeast Queensland region. Despite their higher occurrence in protected areas compared to the other studied macropod species, their future looks even less certain. The projected reduction in suitable habitat under future climate scenarios raises serious concerns, particularly as already fragmented rainforest habitats may become even more isolated. A long‐term study of mammalian assemblages in north Queensland highlights the vulnerability of isolated rainforest habitats, recording significant reductions in species richness and abundance following extensive habitat fragmentation, changes in land use matrix and cyclone disturbances (Laurence et al. 2011). Gradual loss of habitat and fragmentation, particularly in remnant patches of rainforest, threatens the long‐term viability of pademelon species in the Southeast Queensland region due to increased negative impacts of edge effects, invasive species and fire (Mchugh et al. 2022; Smith et al. 2024). Smaller, more isolated populations are at greater risk of a range of negative consequences, including genetic decline, reduced reproductive success, higher mortality rates and increased vulnerability to disease and environmental stochasticity (Frankham 2010; Laurence et al. 2011). These compounded threats may further diminish the species' ability to adapt and survive in a changing climate. The slow progression of habitat changes and the isolation of these rainforest patches and their continuing loss of diversity (Osipova et al. 2017) underline the urgent need for habitat connectivity to allow for habitat progression and range shifts, and increased conservation efforts in Southeast Queensland, especially in regions already experiencing significant environmental stress.

In addition to identifying significant habitat areas within urban regions and projecting substantial habitat declines under climate change, our study also uncovered several other noteworthy findings relevant to conservation planning. The overlapping habitats of eastern grey kangaroos, swamp wallabies and red‐necked wallabies identified in our study could serve as priority areas for multispecies habitat protection. Dietary and temporal partitioning between these species has been demonstrated in many parts of Australia, with sympatric populations well documented (Coulson 1999; Davis et al. 2008; Garnick et al. 2016; Garnick et al. 2018). Therefore, strategic conservation of areas of overlap in significant habitat could play a crucial role in maintaining viable populations of these species while also addressing the broader challenges posed by urban expansion. As a large proportion of this habitat occurs within the urban footprint, urban planning that accounts for the presence and movement patterns of macropods is still essential to mitigate the risks posed by habitat fragmentation and vehicle collisions, which continue to be a significant threat to these species. The incorporation of wildlife inclusive design principles, for example, permeable urban landscapes where wildlife habitat is built into urban design (Apfelbeck et al. 2020), into regional planning instruments such as the Shaping SEQ, Southeast Queensland Regional Plan (Queensland Government 2023), would be a key step in addressing the increasing risk to macropods as urbanisation continues in the decades to come.

The high proportion of suitable habitat overlap between the two pademelon species corroborates with previous research on habitat sympatry between these species (Smith et al. 2022). A large proportion of their significant habitats is in protected areas or on adjacent private lands, supporting the notion of prioritising these areas for proactive conservation, including rehabilitation, exploring the potential for strategic land buy‐back and promoting private landholder conservation efforts. For example, initiatives such as the national Land for Wildlife programme that provides support for landholders to manage wildlife habitat on their properties (including residential or agricultural land uses) could be targeted to assist rehabilitation in high‐priority habitat areas.

## 5 Conclusion

As climate change and urbanisation increasingly shape Australia's landscapes and ecosystems, the future of many macropodid species in Southeast Queensland remains uncertain. This iconic group of animals faces compounded challenges from habitat loss, fragmentation and shifting environmental conditions, all of which threaten their long‐term survival. Our study underscores the urgent need for urgent multifaceted conservation strategies to mitigate these risks. Importantly, the data indicate that all studied macropod species will lose significant amounts of suitable habitat in the Southeast Queensland region under climate change, and that even ‘least concern’ species face prominent threats. For widely distributed species like the eastern grey kangaroo, swamp wallaby and red‐necked wallaby, targeted conservation efforts in the Southeast Queensland region should focus on high suitability areas, especially where their habitats overlap, as these could serve as vital refuges in the face of expanding urban environments. For more at‐risk species, such as the long‐nosed potoroo and black‐striped wallaby, which occupy increasingly restricted habitats, coordinated conservation efforts across state, local and private land tenures are essential. These species' already limited ranges require urgent protection to ensure their persistence. Prioritising areas that are currently underrepresented in conservation actions can make a significant difference in their future survival. Although species like pademelons currently benefit from a higher proportion of protected habitat, the continuing fragmentation and isolation of their environments raise long‐term concerns about their overall viability. The findings of this study highlight the importance of maintaining habitat connectivity and addressing the complex set of factors that could hinder species' adaptability to future environmental changes. The combined pressures of climate change, urbanisation and habitat loss necessitate a broad, adaptive approach to conservation. Collaborative efforts at all levels of governance, integrated urban planning and restoration of critical habitats are essential to safeguard these species as they navigate an unpredictable future in Australia's rapidly changing landscapes. Of note, this study raises the potential reality that populations of these iconic macropods may be severely compromised in the future, meaning that even the ‘least concern’ species should be considered as ‘of concern’ in the Southeast Queensland region.

## Author Contributions

**Elizabeth Ann Brunton:** conceptualization (equal), data curation (equal), formal analysis (equal), funding acquisition (equal), investigation (equal), methodology (equal), project administration (equal), resources (equal), visualization (equal), writing – original draft (lead), writing – review and editing (equal). **Aaron James Brunton:** conceptualization (equal), data curation (equal), formal analysis (equal), investigation (equal), methodology (equal), software (lead), visualization (lead), writing – original draft (equal), writing – review and editing (equal). **Gabriel Conroy:** conceptualization (equal), funding acquisition (equal), methodology (equal), project administration (equal), resources (equal), supervision (equal), writing – original draft (equal), writing – review and editing (equal).

## Conflicts of Interest

The authors declare no conflicts of interest.

## Supporting information


**Table S1:** Summary of model accuracy statistics comparing random forest with MaxEnt for seven macropod species across south‐eastern Queensland, Australia.
**Table S2:** Relative variable importance from random forest models for seven macropod species.
**Figure S1:** Predicted continuous species distributions for the Eastern grey kangaroo ( *Macropus giganteus* ) and Swamp wallaby ( *Wallabia bicolor* ), modelled using random forest. Panels a–b show habitat suitability for the Eastern grey kangaroo: (a) climate refugia projected for 2070 under the moderate representative concentration pathway (RCP) (RCP 4.5) and (b) climate refugia under the high RCP for 2070 (RCP 8.5). Panels c–d depict the same conditions for the Swamp wallaby: (c) climate refugia for 2070 under the moderate RCP (RCP 4.5) and (d) climate refugia under the high RCP (RCP 8.5).
**Figure S2:** Predicted continuous species distributions for the red‐legged pademelon ( *Thylogale stigmatica* ) and red‐necked pademelon ( *Thylogale thetis* ), modelled using random forest. Panels a–b show habitat suitability for the red‐legged pademelon: (a) climate refugia projected for 2070 under the moderate representative concentration pathway (RCP) (RCP 4.5) and (b) climate refugia under the high RCP for 2070 (RCP 8.5). Panels c–d depict the same conditions for the red‐necked pademelon: (c) climate refugia for 2070 under the moderate RCP (RCP 4.5) and (d) climate refugia under the high RCP (RCP 8.5).
**Figure S3:** Predicted continuous species distributions for the red‐necked wallaby (*Notamacropus rufogriseus*) and black‐striped wallaby (*Notamacropus dorsalis*), modelled using random forest. Panels a–b show habitat suitability for the red‐necked wallaby: (a) climate refugia projected for 2070 under the moderate representative concentration pathway (RCP) (RCP 4.5) and (b) climate refugia under the high RCP for 2070 (RCP 8.5). Panels c–d depict the same conditions for the black‐striped wallaby: (c) climate refugia for 2070 under the moderate RCP (RCP 4.5) and (d) climate refugia under the high RCP (RCP 8.5).
**Figure S4:** Predicted continuous species distributions for the long‐nosed potoroo ( *Potorous tridactylus* ) modelled using random forest. (a) Climate refugia projected for 2070 under the moderate representative concentration pathway (RCP) (RCP 4.5) and (b) climate refugia under the high RCP for 2070 (RCP 8.5).

## Data Availability

All files with R code to create the spatial models are available at AaronBrunton/Macropod_climatemodels. Due to the sensitive nature of some occurrence points obtained from private property, location data are available with correspondence from the author.
