# Supplementary material for: From Safe to Stranded: Land Use and Climate Change Threaten Habitat of Iconic Australian Macropods
Source: Ecol Evol. 2025 Oct 9;15(10):e72236. doi: 10.1002/ece3.72236 (PMC12508624; doi:10.1002/ece3.72236)
Supplement: Supplementary file 1 — Table S1: Summary of model accuracy statistics comparing random forest with MaxEnt for seven macropod species across south‐eastern Queensland, Australia. Table S2: Relative variable importance from random forest models for seven macropod species. Figure S1: Predicted continuous species distributions for the Eastern grey kangaroo ( Macropus giganteus ) and Swamp wallaby ( Wallabia bicolor ), modelled using random forest. Panels a–b show habitat suitability for the Eastern grey kangaroo: (a) climate refugia projected for 2070 under the moderate representative concentration pathway (RCP) (RCP 4.5) and (b) climate refugia under the high RCP for 2070 (RCP 8.5). Panels c–d depict the same conditions for the Swamp wallaby: (c) climate refugia for 2070 under the moderate RCP (RCP 4.5) and (d) climate refugia under the high RCP (RCP 8.5). Figure S2: Predicted continuous species distributions for the red‐legged pademelon ( Thylogale stigmatica ) and red‐necked pademelon ( Thylogale thetis ), modelled using random forest. Panels a–b show habitat suitability for the red‐legged pademelon: (a) climate refugia projected for 2070 under the moderate representative concentration pathway (RCP) (RCP 4.5) and (b) climate refugia under the high RCP for 2070 (RCP 8.5). Panels c–d depict the same conditions for the red‐necked pademelon: (c) climate refugia for 2070 under the moderate RCP (RCP 4.5) and (d) climate refugia under the high RCP (RCP 8.5). Figure S3: Predicted continuous species distributions for the red‐necked wallaby (Notamacropus rufogriseus) and black‐striped wallaby (Notamacropus dorsalis), modelled using random forest. Panels a–b show habitat suitability for the red‐necked wallaby: (a) climate refugia projected for 2070 under the moderate representative concentration pathway (RCP) (RCP 4.5) and (b) climate refugia under the high RCP for 2070 (RCP 8.5). Panels c–d depict the same conditions for the black‐striped wallaby: (c) climate refugia for 2070 under the moderate RCP ( [file ECE3-15-e72236-s001.docx]

**Supplementary data**

***From safe to stranded: Land use and climate change threaten habitat of iconic Australian macropods***

**Table S1.** Summary of model accuracy statistics comparing random forest with MaxEnt for seven macropod species across southeastern Queensland, Australia.

| **Species** | **Model** | **AUC** | **COR** | **TSS** | **Deviance** |
| --- | --- | --- | --- | --- | --- |
| Eastern grey kangaroo | rf | 0.94 | 0.71 | 0.75 | 0.32 |
|  | maxent | 0.89 | 0.53 | 0.64 | 0.64 |
| Swamp wallaby | rf | 0.91 | 0.66 | 0.70 | 0.23 |
|  | maxent | 0.89 | 0.43 | 0.64 | 0.58 |
| Red-necked wallaby | rf | 0.90 | 0.65 | 0.65 | 0.30 |
|  | maxent | 0.84 | 0.36 | 0.55 | 0.93 |
| Long-nosed potoroo | rf | 0.97 | 0.69 | 0.87 | 0.03 |
|  | maxent | 0.96 | 0.30 | 0.86 | 0.18 |
| Red-legged pademelon | rf | 0.98 | 0.84 | 0.92 | 0.04 |
|  | maxent | 0.99 | 0.61 | 0.91 | 0.10 |
| Red-necked pademelon | rf | 0.98 | 0.86 | 0.90 | 0.05 |
|  | maxent | 0.99 | 0.78 | 0.89 | 0.06 |
| Black-striped wallaby | rf | 0.85 | 0.52 | 0.60 | 0.05 |
|  | maxent | 0.84 | 0.14 | 0.58 | 0.77 |

**Table S2.** Relative variable importance from random forest models for seven macropod species.

| **Species** | **Metric** | **NDVI** | **Soil** | **Elevation** | **Treecover** | **Max mth Precip** | **Max mth Temp** |
| --- | --- | --- | --- | --- | --- | --- | --- |
| Eastern grey kangaroo | AUC | 7.70% | 2.10% | 19.60% | 1.80% | 17.10% | 3.70% |
|  | COR | 19.30% | 6.10% | 38.90% | 6.60% | 36.50% | 16.10% |
| Swamp wallaby | AUC | 9.00% | 0.80% | 20.10% | 19.90% | 6.90% | 7.70% |
|  | COR | 33.00% | 3.70% | 44.90% | 33.60% | 27.40% | 22.90% |
| Red-necked wallaby | AUC | 9.70% | 1.80% | 16.90% | 9.80% | 13.00% | 22.00% |
|  | COR | 25.20% | 6.00% | 35.10% | 20.90% | 28.90% | 44.60% |
| Long-Nosed potoroo | AUC | 1.20% | 0.10% | 0.30% | 0.60% | 6.80% | 1.50% |
|  | COR | 16.60% | 3.90% | 7.30% | 10.60% | 54.40% | 21.50% |
| Red-legged pademelon | AUC | 0.20% | 0.20% | 0.20% | 1.40% | 4.60% | 3.80% |
|  | COR | 3.80% | 7.20% | 7.30% | 8.00% | 37.00% | 40.30% |
| Red-necked pademelon | AUC | 0.70% | 0.10% | 0.30% | 0.90% | 4.60% | 2.10% |
|  | COR | 5.60% | 1.00% | 7.00% | 9.60% | 39.20% | 30.80% |
| Black-striped wallaby | AUC | 0.70% | 0.10% | 0.30% | 0.90% | 4.60% | 2.10% |
|  | COR | 14.00% | 3.00% | 22.80% | 50.60% | 64.60% | 11.00% |


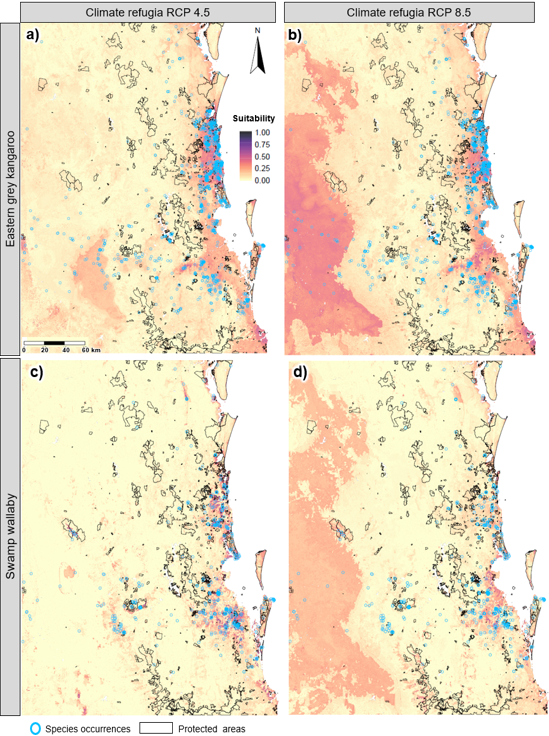


**Fig S1.** Predicted continuous species distributions for the Eastern grey kangaroo (*Macropus giganteus*) and Swamp wallaby (*Wallabia bicolor*), modelled using random forest. Panels a-b show habitat suitability for the Eastern grey kangaroo: (a) climate refugia projected for 2070 under the moderate representative concentration pathway (RCP) (RCP 4.5), and (b) climate refugia under the high RCP for 2070 (RCP 8.5). Panels c-d depict the same conditions for the Swamp wallaby: (c) climate refugia for 2070 under the moderate RCP (RCP 4.5), and (d) climate refugia under the high RCP (RCP 8.5).

**
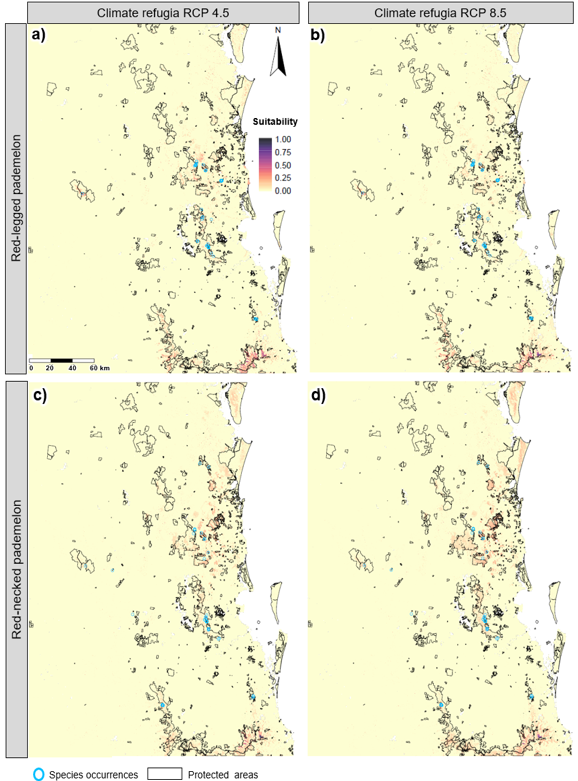
Fig S2.** Predicted continuous species distributions for the Red-legged pademelon (*Thylogale stigmatica*) and Red-necked pademelon (*Thylogale thetis*), modelled using random forest. Panels a-b show habitat suitability for the Red-legged pademelon: (a) climate refugia projected for 2070 under the moderate representative concentration pathway (RCP) (RCP 4.5), and (b) climate refugia under the high RCP for 2070 (RCP 8.5). Panels c-d depict the same conditions for the Red-necked pademelon: (c) climate refugia for 2070 under the moderate RCP (RCP 4.5), and (d) climate refugia under the high RCP (RCP 8.5).


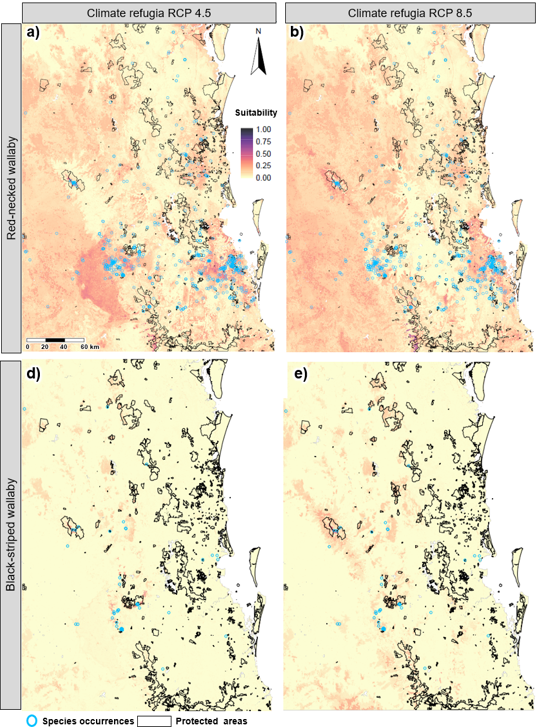


**Fig S3.** Predicted continuous species distributions for the Red-necked wallaby (*Notamacropus rufogriseus*) and Black-striped wallaby (*Notamacropus dorsalis*), modelled using random forest. Panels a-b show habitat suitability for the Red-necked wallaby: (a) climate refugia projected for 2070 under the moderate representative concentration pathway (RCP) (RCP 4.5), and (b) climate refugia under the high RCP for 2070 (RCP 8.5). Panels c-d depict the same conditions for the Black-striped wallaby: (c) climate refugia for 2070 under the moderate RCP (RCP 4.5), and (d) climate refugia under the high RCP (RCP 8.5).


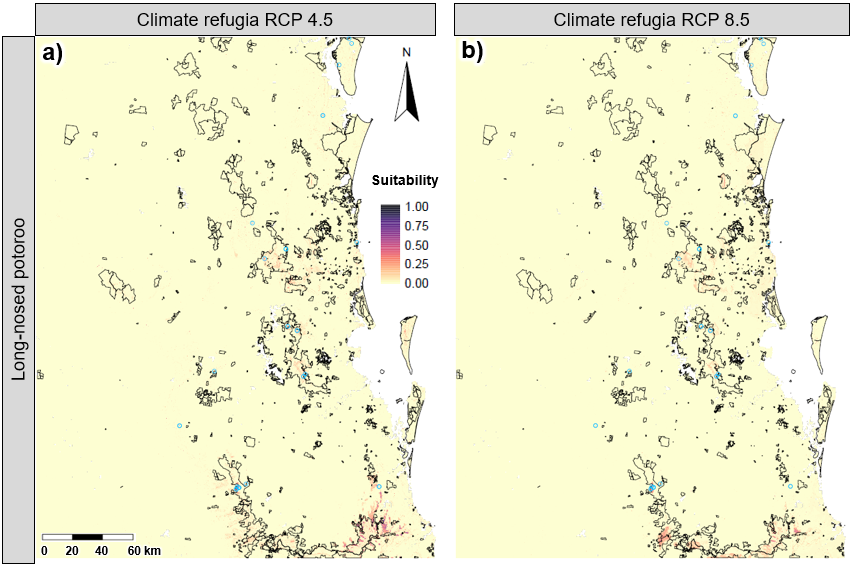


**Fig S4.** Predicted continuous species distributions for the Long-nosed potoroo (*Potorous tridactylus*) modelled using random forest. Panel (a) shows climate refugia projected for 2070 under the moderate representative concentration pathway (RCP) (RCP 4.5), and (b) climate refugia under the high RCP for 2070 (RCP 8.5).
